# A 16S Next Generation Sequencing Based Molecular and Bioinformatics Pipeline to Identify Processed Meat Products Contamination and Mislabelling

**DOI:** 10.3390/ani12040416

**Published:** 2022-02-10

**Authors:** Nyaradzo Stella Chaora, Khulekani Sedwell Khanyile, Kudakwashe Magwedere, Rian Pierneef, Frederick Tawi Tabit, Farai Catherine Muchadeyi

**Affiliations:** 1 Department of Life and Consumer Sciences, College of Agriculture and Environmental Sciences, University of South Africa, Rooderpoort 1709, South Africa; nyarry.stch@gmail.com (N.S.C.); Tabitft@unisa.ac.za (F.T.T.); 2 Biotechnology Platform, Agricultural Research Council, Private Bag X 05, Onderstepoort, Pretoria 0110, South Africa; kskhanyile@gmail.com (K.S.K.); PierneefR@arc.agric.za (R.P.); 3 Directorate of Veterinary Public Health, Department of Agriculture, Land Reform and Rural Development, Pretoria 0001, South Africa; KudakwasheM@dalrrd.gov.za

**Keywords:** processed meat, adulteration, mitochondrial 16S rRNA gene, Next-Generation Sequencing

## Abstract

**Simple Summary:**

Meat adulteration and fraud encompasses the deliberate fraudulent addition or substitution of proteins of animal or plant origin in edible products primarily for economic gain. The mitochondrial 16S ribosomal (rRNA) gene was used to identify species that are present in pure and processed meat samples. The meat samples were sequenced using an Illumina sequencing platform, and bioinformatics analysis was carried out for species identification. The results indicated that pork was the major contaminant in most of the meat samples. The bioinformatics pipeline demonstrated its specificity through identification of species specific and quantification of the contamination levels across all samples. Food business operators and regulatory sectors can validate this method for food fraud checks and manage any form of mislabeling in the animal or plant protein food ecosystem.

**Abstract:**

Processed meat is a target in meat adulteration for economic gain. This study demonstrates a molecular and bioinformatics diagnostic pipeline, utilizing the mitochondrial 16S ribosomal RNA (rRNA) gene, to determine processed meat product mislabeling through Next-Generation Sequencing. Nine pure meat samples were collected and artificially mixed at different ratios to verify the specificity and sensitivity of the pipeline. Processed meat products (n = 155), namely, minced meat, biltong, burger patties, and sausages, were collected across South Africa. Sequencing was performed using the Illumina MiSeq sequencing platform. Each sample had paired-end reads with a length of ±300 bp. Quality control and filtering was performed using BBDuk (version 37.90a). Each sample had an average of 134,000 reads aligned to the mitochondrial genomes using BBMap v37.90. All species in the artificial DNA mixtures were detected. Processed meat samples had reads that mapped to the *Bos* (90% and above) genus, with traces of reads mapping to *Sus* and *Ovis* (2–5%) genus. Sausage samples showed the highest level of contamination with 46% of the samples having mixtures of beef, pork, or mutton in one sample. This method can be used to authenticate meat products, investigate, and manage any form of mislabeling.

## 1. Introduction

Meat species identification is a subject that has received special attention worldwide, mainly due to the increased incidence of fraudulent practices that have been reported [1,2,3,4]. These reports have led to consumers demanding the accurate identification and labelling of meat products [5]. Incidents of meat species substitution include contamination of a product with a cheaper-priced protein. For instance, replacing Grade A beef with rejected horse meat, replacing mutton with a lower grade of beef, or replacing mutton with pork [3,4]. The addition of plant proteins, such as grain by-products or soyabeans, to meat products like beef patties and sausages has also been reported [6]. Meat species substitution is common in processed meat products that are difficult to accurately identify morphologically once processed into value-added products. For instance, pork is intentionally added to beef products to reduce production costs [7]. Once the two different meats are minced or ground it is difficult to identify them using the naked eye. Meat adulteration predominately occurs in ground meat products [8].

Consumers have a right to purchase meat products that are correctly labelled for reasons of health (allergies), religious belief, individual preference and ethics [9]. Therefore, there is need for the accurate identification of meat species in processed meat products. Food labelling regulations require that ingredients in food products are accurately declared to consumers [1]. The governing organizations in South Africa have issued new legislation to encourage clarity and the accurate explanation of food products, in response to consumer demand. These are the controls linked to Advertising and Labelling of Foodstuffs (R. 146/2010) comprised of a compulsory ingredient list on food labels [10] and the Consumer Protection Act (R. 147/2009), which prevents unfair marketing and business practices and provides an improved standard of consumer information [11]. The food regulation standard in Europe requires that meat products should be accurately labeled with information that includes the composition and percentage of ingredients included in the products [12].

Techniques for meat species identification need to be reliable, rapid and cheap enough for routine applications. In the past, the identification of meat species has been conducted using protein-based methods, which entail different immunological, chromatographic, and electrophoretic methods [4,13]. However, the disadvantages of protein-based methods are that proteins are denatured by heat, salt and pressure, making protein-based methods unsuitable for the identification of species in seasoned, cured or dried meat, and meat patties [14,15]. Protein-based methods are also inaccurate in identifying species that have a close phylogenetic relationship due to cross-reactivity, for instance in poultry species [1,13,16,17] deoxyribonucleic acid- (DNA) based methods are now preferred in place of protein-based methods, because DNA is more stable during heating and less likely to be disturbed during food processing [13].

Mitochondrial DNA (mtDNA) is commonly used in the identification of meat species, as it can be extracted, undamaged, from cooked and processed meat products [18,19]. Mitochondrial DNA is commonly used in species identification, since mtDNA occurs in multiple copies (an average of 1000 per cell), can withstand heat, salt, and pressure and can discriminate closely related species due to its high rate of evolution [20]. Several mtDNA genes have been used in meat species identification, such as cytochrome b, cytochrome c oxidase subunit 1 (COI), NADH dehydrogenase subunit 2 and 5 (ND2 and ND5), ATPase 6 and 8, mitochondrial 16S, and 12S ribosomal RNA (rRNA) genes [21]. Mitochondrial DNA-based methods that have been used for species identification in the past are polymerase chain reaction (PCR) [22], PCR-restriction fragment length polymorphism (PCR-RFLP) [23], species-specific PCR [24,25], DNA hybridization [17], multiplex PCR [26], and real-time PCR [17,27]. The limitations of previous DNA-based methods are that species-specific PCR methods were used and, therefore, these studies targeted specific species as opposed to having a universal method that targets any species. Species-specific methods are advantageous when species in a sample are known, however, a universal method is a better approach for investigating multiple and unsuspected contaminations in meat products. Recently, there have been advances in DNA-based methods, specifically in DNA sequencing technologies. Next-Generation Sequencing (NGS) is a method that can generate sequenced data from degraded DNA, and one that can produce large amounts of sequenced data at a low cost and with minimum errors [28]. Furthermore, essentially no prior information of species is needed, making NGS technology a non-species-specific method [28]. With the NGS method, mtDNA barcoding genes can be sequenced using universal primers, without knowledge of which species are present and without targeting specific species. This, then, enables the identification of every species present in a sample, as against merely the suspected/hypothesised ones [17]. However, the database used in the identification of species needs to be comprehensive, such that it contains a large number of species to enable accurate identification.

Meat species contamination has been reported in the South African meat sector [1,2,29]. A routine universal diagnostic method that can be used by laboratories needs to be developed, as the methods used to date, in South Africa, have been species-specific. Meat producers can use this method to authenticate their products and gain consumers’ confidence in the products they will purchase. Mitochondrion carries extra-chromosomal genetic material and contains high copy numbers as compared to single copy nuclear genes. Therefore, mitochondrial DNA is the preferred analytical tool in forensic, molecular, and zoological experiments. The objective of this study was, therefore, to develop a universal and robust diagnostic molecular and bioinformatics pipeline that can utilize the mitochondrial 16S ribosomal RNA (rRNA) barcoding gene to identify processed meat product mislabeling/contamination using NGS. Universal mitochondrial 16S rRNA primers will be used in this study to identify different species, including those in mixed samples. Meat suppliers can possibly implement the current method to authenticate their products, and the food industry may also use this method to reveal any form of mislabeling that may be present.

## 2. Materials and Methods

### 2.1. Collection of Pure Meat Samples to Confirm the Reliability of the Species Identification Pipeline

Pure meat samples were collected as controls to verify the use of the 16S rRNA gene in the molecular and bioinformatics pipeline developed. This was done primarily to test the specificity, sensitivity, and ability of the pipeline to be used as a diagnostic method. Nine unprocessed pure meat samples from nine different species were collected from a local butchery in Pretoria (South Africa), transported in an icebox and stored at −20 °C. The pure meat samples were placed in separate plastics upon collection, transportation, and storage, to avoid any unintentional cross-contamination. These species were pig, cattle, sheep, chicken, turkey, goat, ostrich, duck, and kangaroo.

### 2.2. The Collection of Processed Meat Samples for Species Identification

Meat products were randomly collected from processing plants and retail outlets in the Gauteng and Free State provinces in South Africa for the species identification test. A total of 155 samples from the meat value chain were collected and analyzed. Four different categories of processed meat products were collected for analysis, namely, minced meat (49), burger patties (35), biltong (28), and raw sausages (43). Some samples included information on which species they were produced from, and, of these, 22 were beef mince, 20 were beef patties, 17 were beef biltong, and 21 were beef sausages. All samples were transported in an ice box and stored at −20 °C.

### 2.3. DNA Extraction

Genomic DNA from the pure meat samples used for the verification test was ex-tracted manually from 40 mg of each meat sample. A Macherey–Nagel NucleoMag Tissue kit for DNA purification from cells and tissue (Macherey–Nagel, Düren, Germany) was used for DNA extraction according to the Genomic DNA from Tissue user manual. The pure DNA was stored at −20 °C while awaiting further analysis. Thirteen two-species DNA mixtures of known species and composition were artificially mixed (Table 1). Two ratios were used for the DNA mixtures, 1:1 (50%:50%) and 0.9:0.1 (90%:10%). The artificially mixed samples were used to test the specificity of the 16S universal primers, by confirming the origin of the known species in the 1:1 ratio mixture. The ratio of 0.9:0.1 was used to test the sensitivity of the pipeline, using, as a metric thereof, the smallest amount of DNA that the pipeline could correctly identify at an affordable cost. Each mixed ration had three replicates. The concentration of the DNA of each pure meat sample used was normalized to two different concentrations, 25 ng/µL and 5ng/µL prior to running of the PCR. The differences in DNA concentration are explained in the PCR step. Genomic DNA for the species identification of the samples collected from the meat value chain was extracted from 300 mg of each processed meat sample, using a Hamilton Microlab Star automated liquid handler (Hamilton Inc., Reno, NV, USA). A Macherey–Nagel NucleoMag Tissue kit for DNA purification from cells and tissue (Macherey–Nagel, Düren, Germany) was used for DNA extraction according to the Genomic DNA from Tissue user manual. The DNA concentration of the meat value chain samples was between 28–467 ng/µL prior to PCR testing. The quantification of DNA for all samples was checked using the Qubit^®^ fluorescent dye method, and gel electrophoresis was used to assess the quality of the starting material. A ratio of A260/A280 was used to access the purity of all extracted DNA.

### 2.4. PCR Amplification of the Mitochondrial 16S rRNA Gene

Polymerase chain reaction (PCR) for the mitochondrial 16S rRNA gene was performed using universal mammalian primers designed by [17] and tailed with Nextera adapters (Table 2). Thermal cycling was performed in a Labnet MultigeneTM Gradient Thermal Cycler (Woodridge, IL, USA) at a final volume of 50 µL. All the ratio mixtures had a normalized DNA concentration of 50 ng/µL in the PCR run. This was done so that if one ratio mixture did not identify the contained species, it would not be due to the DNA having a lower concentration. The PCR for the 50%:50% (1:1) ratio mixture contained 25 µL of 2× Hot start PCR mastermix, 5 µL of each forward and reverse primer (1 mM final concentration), 13 µL RNase-free water and 1 µL of 25 ng/µL DNA template of each species. This brought the total amount of DNA template for the 50%:50% ratio mixture to 2 µL and the concentration to 50 ng/µL. The PCR for the 90%:10% (0.9:0.1) ratio mixture contained 25 µL Kapa HiFi Hotstart Readymix (Roche, NY, USA), 5 µL of each forward and reverse primer (1 mM final concentration), 12.2 µL RNase-free water, 1.8 µL of the 25 ng/µL DNA template for the species with a ratio of 90% and 1 µL of 5 ng/µL DNA template for the species with a ratio of 10%. This brought the total amount of DNA template for the 90%:10% ratio mixture to 2.8 µL and the concentration to 50 ng/µL. Similar to the DNA extraction process, sterile tips and PCR tubes were not reused and the pipettes and work-bench area were disinfected with 70% ethanol between analyses.

The PCR for the samples from the meat value chain contained 25 µL of Kapa HiFi Hotstart Readymix (Roche, NY, USA), 5 µL of each forward and reverse primer (1 mM final concentration), 13 µL RNase-free water, and 2 µL of DNA template. The PCR conditions for all samples were as follows: denaturation at 95 °C for 3 min, followed by 30 cycles of 90 °C for 20 s, 65 °C for 30 s, 72 °C for 30 s, and finalization at 72 °C for 5 min. The PCR products for the mitochondrial 16S rRNA gene were 186 bp in length. The PCR products were viewed in 2% agarose gels in 1× tris-acetate-EDTA (TAE) buffer at 90V for 45 min. The amplified products were visualized under ultra-violet light in a transilluminator. Purification of PCR products was performed using a Qiagen MiniElute^®^ PCR purification kit (Qiagen, Germany) according to the manufacturer’s protocol. Quantification of the purified samples was done using the Qubit^®^ fluorescent dye method. A ratio of A260/A280 was used to access the purity of all extracted DNA. The purified products were stored at 4 °C prior to sequencing.

### 2.5. Library Preparation and Illumina MiSeq Sequencing

Prior to sequencing, library preparation was performed using the 16S Meta-genomics Sequencing Library Preparation kit, according to the manufacturer’s protocol (Illumina, Inc, San Diego, CA, USA). Quality control of the sample library and quantification of the DNA library templates was performed. Quantification of DNA was done using Qubit^®^ fluorescent dye method. The library size distribution was checked using a High Sensitivity DNA chip. Thereafter, the indexed libraries were normalized, pooled and loaded onto an Illumina MiSeq reagent cartridge using MiSeq reagent kit v3 and 600 cycles. The paired end 2 × 300 bp sequencing was run on an Illumina MiSeq sequencer at 0.2 × coverage at the Biotechnology Platform, Agricultural Research Council, Onderstepoort, South Africa. The DNA from pure meat samples were each sequenced individually prior to artificially mixing the DNA, to confirm the origin of each meat type.

### 2.6. Bioinformatics and Data Analyses

Prior to species identification, quality control, adapter removal, decontamination, and error correction of the raw sequence data was done using BBDuk (version 37.90; https://jgi.doe.gov/data-and-tools/bbtools/bb-tools-user-guide/bbduk-guide/, accessed on 20 July 2020). All available mitochondrial genomes (10,788) were downloaded from the NCBI RefSeq database (https://ftp.ncbi.nlm.nih.gov/refseq/release/mitochondrion, accessed on 20 July 2020) [30]. Filtered reads were aligned to the complete mitochondrial genomes using BBMap v37.90 [31], (https://www.osti.gov/biblio/1241166-bbmap-fast-accurate-splice-aware-aligner, accessed on 20 July 2020) for species identification and the average fold coverage across a mitochondrial reference genome was used for further analysis. The average fold of each sample was exported into Microsoft Excel to calculate the percentage of the number of reads that aligned to a reference, over how much of the reference was covered in a sample. The percentage average fold was used to determine samples that were contaminated and uncontaminated.

**Figure 1:**
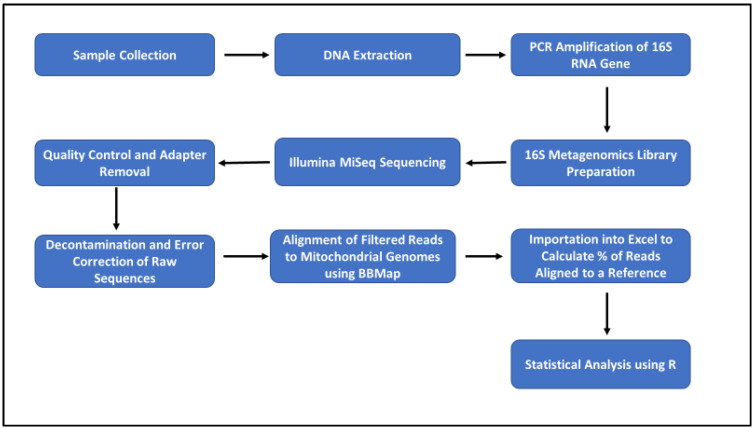
Workflow for molecular and bioinformatics pipeline for species identification.

Statistical analysis was initially performed on the pure and artificially mixed samples using R software, to determine whether our pipeline would work in a controlled environment. The percentage deviation from the expected composition within the pure (100%) and artificially mixed (50%:50% and 90%:10%) samples was also determined, and these values were used to calculate the mean (using absolute values), median, standard deviation, and variance. Bar plots, plotting the percentage composition by species present in all samples, were constructed, showing samples that were contaminated and uncontaminated. A chi-square proportion test was used to determine whether there was a significant correlation between two categorical variables, i.e., contaminated and uncontaminated meat samples. A *p*-value was determined as a result of the number of contaminated versus uncontaminated. Associations between contaminated versus uncontaminated meat were considered statistically significant only for *p*-values ≤ 0.05. Cramer’s V test, which measures how strongly two categorical fields are associated, was also performed. The confidence interval (CI) was set at 95% and the number of samples observed (n_obs_) was also determined. The workflow of the molecular and bioinformatics pipeline is shown in Figure 1.

## 3. Results

### 3.1. Identification of Meat Species Using Pure DNA from Known Meat Types

**Figure 2:**
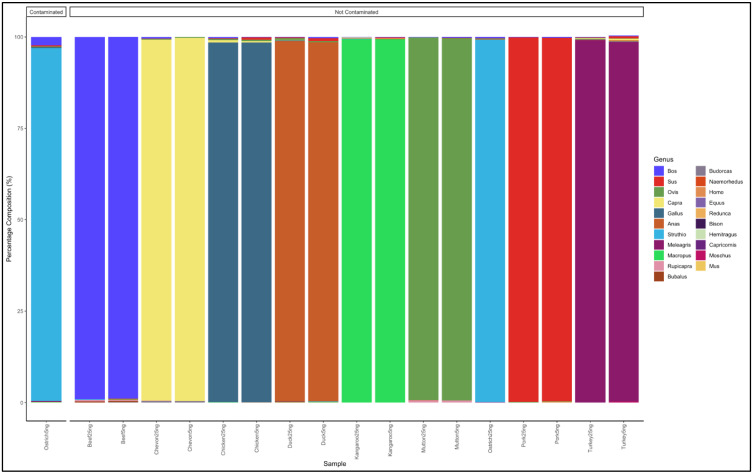
Percentage average fold of pure DNA from known meat types.

Paired-end reads, with a length of 300 bp, were sequenced using the Miseq sequencer. Each sample had an average of 156,863 reads, before quality filtering, and 134,230 reads after quality filtering. Nine pure meat samples with two replicates each were analysed, for a total of 18 analysed pure samples. The reads obtained mapped to the corresponding pure meat species, with a similarity of 98% and above for all meat types (Figure 2). Besides identifying the expected genera, traces of other meat species were observed (Table S1). The beef, mutton, and pork meat samples had reads with an average fold of 99% for the *Bos*, *Ovis*, and *Sus* genus, respectively. The chevon, chicken, and duck meat samples had reads with an average fold of 98% for the *Capra*, *Gallus*, and *Anas* genus, respectively. The turkey and kangaroo meat samples had reads with an average fold of 99% for the *Meleagris*, *Struthio*, and *Macropus* genus, respectively. One of the ostrich samples was contaminated with beef, as it showed a proportion with reads that mapped to the *Bos* genus (Figure 1). Based on these results from the controls, samples whose highest percentage average fold was less than 98% were considered contaminated.

### 3.2. Identification of Meat Species Using Pure DNA from Known Meat Types Artificially Mixed at a Ratio of 1:1

**Figure 3:**
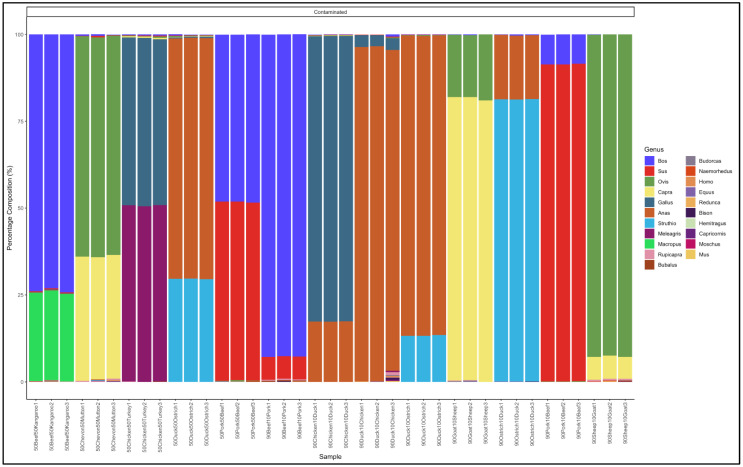
Percentage of average fold of pure DNA from two known meat types artificially mixed at a ratio of 1:1 (50%:50%).

The pipeline identified all the meat types whose DNA were in the 1:1 ratio mixture. However, some positive and/or negative deviations were observed from the expected 50:50 percentages (Table S2). The beef (50%) and kangaroo (50%) DNA mixtures had reads with an average fold of 73% and 25% for the *Bos* (cattle) and *Macropus* (kangaroo) genus, respectively (Figure 3). The chevon (50%) and mutton (50%) DNA mixtures had reads with an average fold of 35% and 63% for the *Capra* (goat) and *Ovis* (sheep) genus, respectively. The chicken (50%) and turkey (50%) DNA mixtures had reads with an average fold of 48% and 50% for the *Gallus* (chicken) and *Meleagris* (turkey) genus, respectively.

The duck (50%) and ostrich (50%) DNA mixtures had reads with an average fold of 69% and 29% for the *Anas* (duck) and *Struthio* (ostrich) genus, respectively. The pork (50%) and beef (50%) DNA mixtures had reads with an average fold of 51% and 48% for the *Sus* and *Bos* genus, respectively. The greatest deviations for the 1:1 (50% of each species) ratio were between beef (*Bos*): kangaroo (*Macropus*) where beef meat had an overestimation of 23% and kangaroo meat had an underestimation of 24%. The lowest deviations were between pork (*Sus*): beef (*Bos*), where pork had an overestimation of 1.5% and beef had an underestimation of 1.9% (Table S2).

### 3.3. Identification of Pure DNA from Known Meat Types Artificially Mixed at a Ratio of 9:1

The pipeline showed that all the meat types whose DNAs were in the 9:1 ratio mixture were identified. Some positive and/or negative deviations were observed from the expected 90:10 percentage ratio (Table S3). The beef (90%) and pork (10%) DNA mixtures had reads with an average fold of 92% and 6% for the *Bos* (cattle) and *Sus* (pig) genus, respectively (Figure 3). The chicken (90%) and duck (10%) DNA mixtures, the reads had an average fold of 82% and 17% for the *Gallus* (chicken) and *Anas* (duck) genus, respectively. The duck (90%) and chicken (10%) DNA mixtures had reads with an average fold of 96% and 3% for the *Anas* (duck) and *Gallus* (chicken) genus, respectively. The duck (90%) and ostrich (10%) DNA mixtures had reads with an average fold of 86% and 13% for the *Anas* (duck) and *Struthio* (ostrich) genus, respectively. The goat (90%) and sheep (10%) DNA mixtures had reads with an average fold of 81% and 17% for the *Capra* (goat) and *Ovis* (sheep) genus, respectively. The ostrich (90%) and duck (10%) DNA mixtures had reads with an average fold of 81% and 18% for the *Struthio* (ostrich) and *Anas* (duck) genus, respectively. The pork (90%) and beef (10%) DNA mixtures had reads with an average fold of 91% and 8% for the *Sus* (pig) and *Bos* (cattle) genus, respectively. The mutton (90%) and chevon (10%) DNA mixtures had reads with an average fold of 92% and 6% for the *Ovis* (sheep) and *Capra* (goat) genus, respectively. The lowest deviations for the 9:1 (90% and 10%) of each species ratio were between pork (*Sus*): beef (*Bos*). Pork meat had an overestimation of 1.2% and beef meat had an underestimation of 1.5%. The greatest deviations were between ostrich (*Struthio*): duck (*Anas*). Ostrich meat had an underestimation of 8.8% and duck meat had an overestimation of 8.5% (Table S3).

### 3.4. Percentage Deviation from Expected Percentages in the Pure and Artificially Mixed Samples

The percentage deviation (expected–observed) was determined and used to calculate the descriptive statistics (mean, median, standard deviation, variance, minimum value, and maximum value) of the pure and artificially mixed samples (Table 3). Measures of central tendency were described by the mean and median values, while measures of variability were described by standard deviation, variance, and minimum and maximum values. The range within the artificially mixed samples was 23.59% and 3.19% for the pure samples. The higher spread of values in the artificially mixed samples lead to a higher mean value, and that, in turn, resulted in a higher standard deviation and variance that was further from zero. In contrast, the smaller spread of values within the pure samples lead to a lower mean value that resulted in a lower standard and variance that was closer to zero.

### 3.5. Identification of Species and Species Contamination of Processed Meat Collected from Processing Plants and Retail Outlets

#### 3.5.1. Biltong

**Figure 4:**
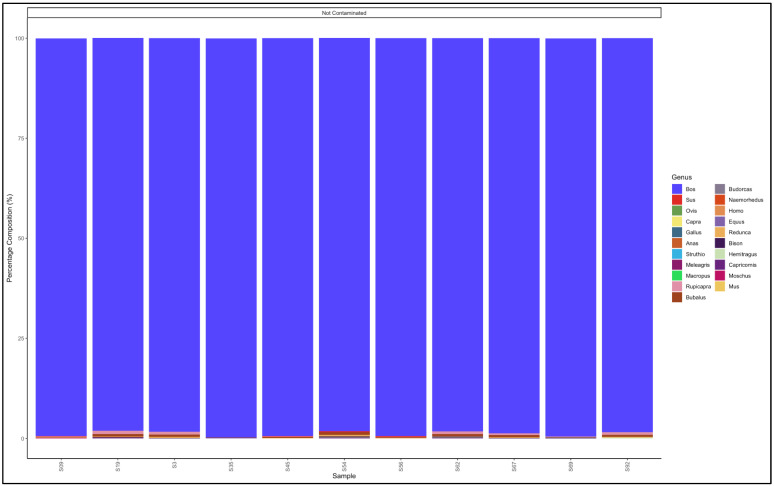
Percentage composition of biltong samples not specified which species they are from.

The pipeline demonstrated that all the 11 biltong samples that had not specified from which species they were from were uncontaminated and were essentially beef (*Bos* genus) (Figure 4). The biltong samples, however, contained trace contaminants of other species (Table S4).

**Figure 5:**
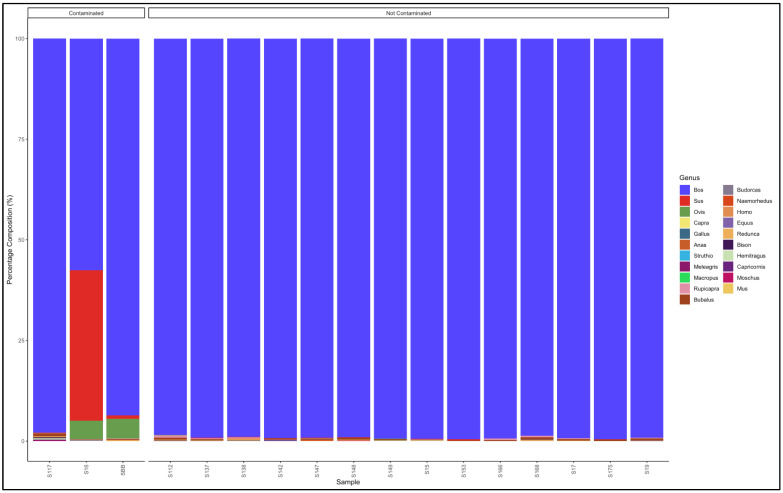
Percentage composition of samples labeled as beef biltong.

The pipeline also demonstrated that 14 out of the 17 samples labelled as beef biltong were uncontaminated and were also essentially from beef (*Bos* genus) (Figure 5). The contaminated samples, descending from most contaminated, were predominantly composed as follows: Sample 16: *Bos* (cattle) (57.5) and *Sus* (pig) (37.4); Sample S: *Bos* (cattle) (93.6) and *Ovis* (sheep) (4.9); and Sample 117: *Bos* (cattle) (97.9) and *Bubalus* (Buffalo) (0.7). The major contaminants of the labelled beef biltong products were pork (*Sus*) and mutton (*Ovis*). Sample 16 contained beef (*Bos*) (57.5) but was contaminated with pork (*Sus*) (37.4) and mutton (*Ovis*) (4.6) (Table S5).

#### 3.5.2. Mince

**Figure 6:**
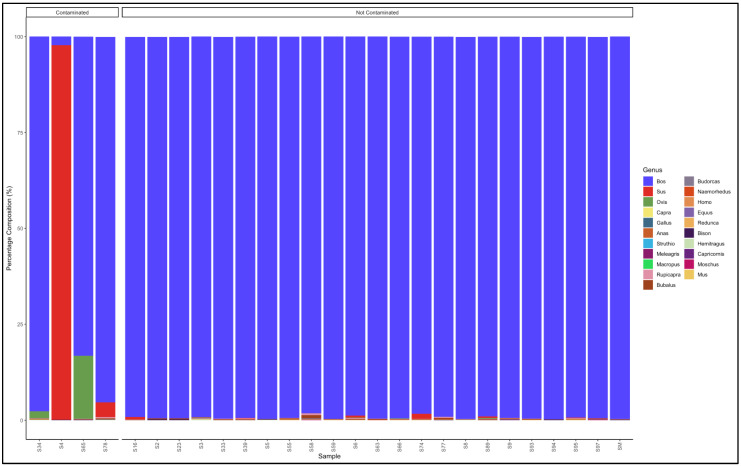
Percentage composition of mince samples that had not specified which species they were from.

The pipeline demonstrated that 23 out of 27 mince samples that had not specified from which species they originated were uncontaminated and were essentially from beef (*Bos* genus) (Figure 6). The five contaminated samples, descending from the most contaminated were predominantly composed as follows: Sample 65: *Bos* (cattle) (83,2) and *Ovis* (sheep) (16.4); Sample 78: *Bos* (cattle) (95,3) and *Sus* (pig) (3.8); Sample 4: *Sus* (pig) (97.7) and *Bos* (cattle) (2.2); and Sample 34: *Bos* (cattle) (97.7) and *Ovis* (sheep) (1,8). Sample 4 was evidently pork mince (predominantly Sus genus) contaminated with beef (*Bos* genus), while the rest were beef (*Bos* genus) mince contaminated with either pork (*Sus* genus) or mutton (*Ovis* genus) (Table S6).

**Figure 7:**
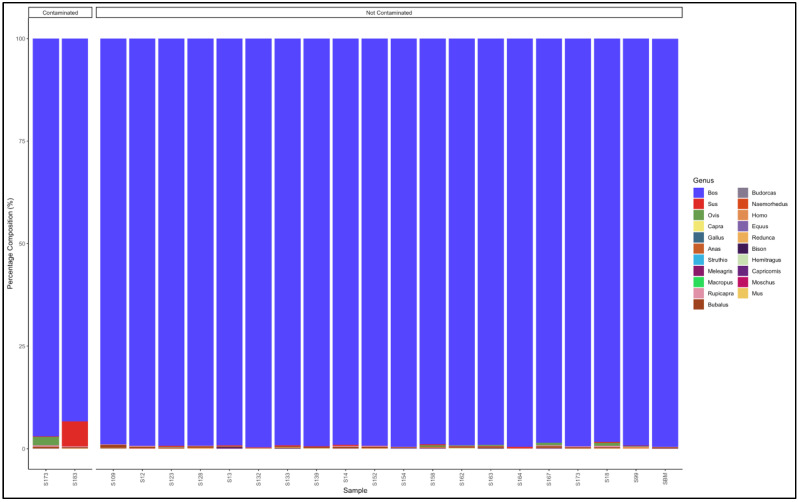
Percentage composition of samples labeled as beef mince.

The pipeline also demonstrated that 20 out of the 22 samples labelled as beef mince were uncontaminated and were essentially from beef (*Bos* genus) (Figure 7). The contaminated descending from the most contaminated were predominantly composed as follows: Sample 183: *Bos* (cattle) (93.3) and *Sus* (pig) (6.1); and Sample 17: *Bos* (cattle) (97.0) and *Ovis* (sheep) (2.0). The major contaminants of the labelled beef mince products were pork (*Sus*) and mutton (*Ovis*). Two samples, S158 and S99 had traces (0.1% and 0.5%, respectively) of the *Homo* (human) genus (Table S7).

#### 3.5.3. Patties

**Figure 8:**
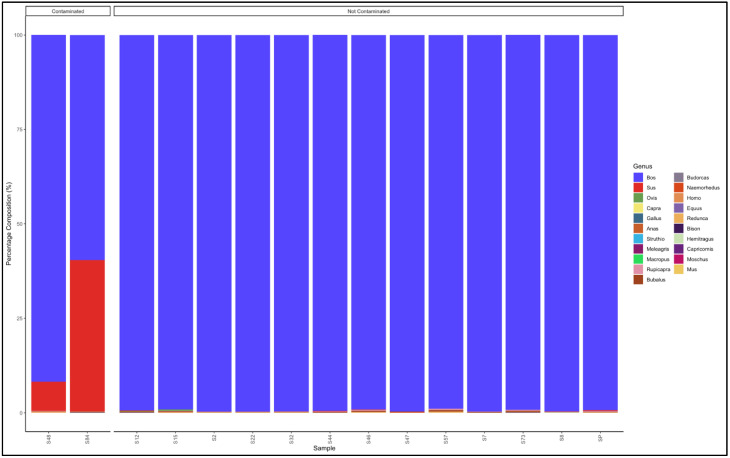
Percentage composition of patty samples not specified which species they are from.

The pipeline demonstrated that 13 out of the 15 patty samples that had not specified which species they are from were uncontaminated and were essentially from beef (*Bos* genus) (Figure 8). The two contaminated samples, descending from the most contaminated, were predominantly composed as follows: Sample 15: *Sus* (pig) (59.6) and *Bos* (cattle) (40.0), and Sample 48: *Bos* (cattle) (91.8) and *Sus* (pig) (7.7). Sample 15 was a pork patty (*Sus* genus) contaminated with beef (*Bos* genus), while sample 48 was a beef (*Bos* genus) patty contaminated with pork (*Sus* genus) (Table S8).

**Figure 9:**
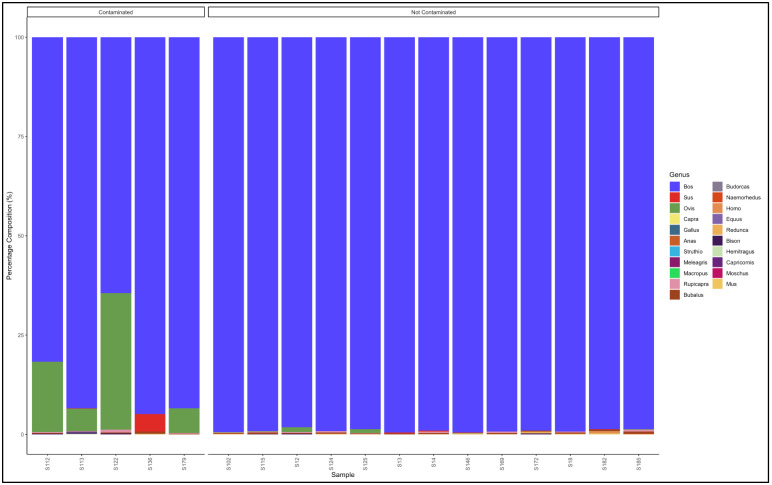
Percentage composition of samples that were labeled as beef patty.

The pipeline also demonstrated that 13 out of 18 samples labelled as beef patty were uncontaminated and were essentially from beef (*Bos* genus) (Figure 9). The contaminated samples descending from the most contaminated were predominantly composed as follows: Sample 122: *Bos* (cattle) (64.5), *Ovis* (sheep) (34.3); Sample 112: *Bos* (cattle) (81.7) and *Ovis* (sheep) (17.7); Sample 179: *Bos* (cattle) (93.4) and *Ovis* (sheep) (6.2); Sample 113: *Bos* (cattle) (93.4) and *Ovis* (sheep) (5.6); and Sample 136: *Bos* (cattle) (94.9) and *Sus* (pig) (4.4). The major contaminants of the labelled beef patty products were mutton (*Ovis*) and pork (Sus). Sample 122 contained beef (*Bos*) (64.5) but was contaminated with mutton (*Ovis*) (34.3) (Table S9).

#### 3.5.4. Sausages

**Figure 10:**
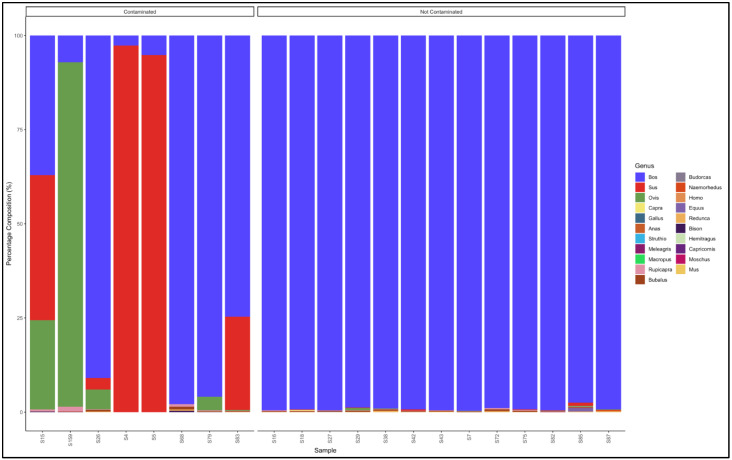
Percentage composition for sausage samples not specified which species they are from.

The pipeline demonstrated that 14 out of the 21 sausage samples that had not specified which species they were from were uncontaminated and were essentially from beef (*Bos* genus) (Figure 10).

The seven contaminated samples descending from the most contaminated were predominantly composed as follows: Sample 15: *Bos* (cattle) (37.1) *Sus* (pig) (38.5) and *Ovis* (sheep) (23,7); Sample 83: *Bos* (cattle) (74.7) and *Sus* (pig) (24.7); Sample 26: *Bos* (cattle) (91.0), *Ovis* (sheep) (5.2) and *Sus* (pig) (3.0); Sample 159: *Ovis* (sheep) (91.4) and *Bos* (cattle) (7.0); Sample 5: *Sus* (pig) (94,8) and *Bos* (cattle) (5,2); Sample 79: *Bos* (cattle) (95.9) and *Ovis* (sheep) (3.6); Sample 4: *Sus* (pig) (97.4) and *Bos* (cattle) (2.7); and Sample 68: *Bos* (cattle) (97.9), *Bubalus* (buffalo) (0.8) and *Rupicapra* (goat antelope) (0.6). Sample 15 was a mixed sausage made up of a substantial amount of beef (*Bos*), pork (*Sus*) and mutton (*Ovis*). Sample 159 was a mutton sausage contaminated with beef (*Bos* genus), while sample 4 and 5 were pork (*Sus* genus) sausages contaminated with beef (*Bos* genus (Table S10).

**Figure 11:**
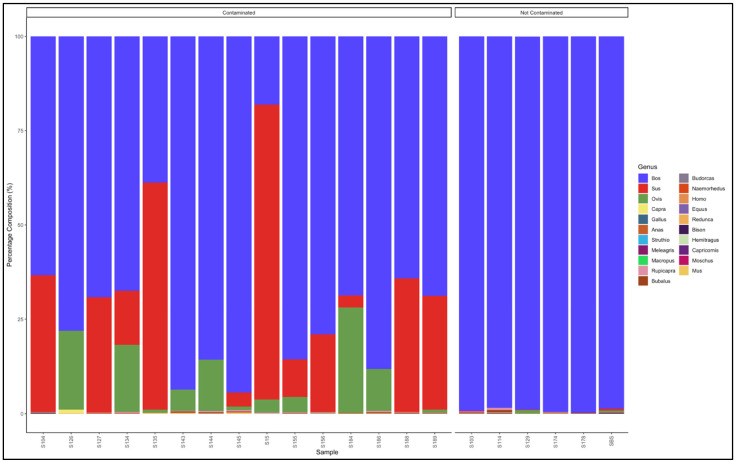
Percentage composition of samples labeled as beef sausages.

The pipeline also demonstrated that 7 out of 21 (33%) samples labelled as beef sausage were uncontaminated and were essentially from beef (*Bos* genus) (Figure 11). The major contaminants of labelled beef sausage products were mutton (*Ovis*) and pork (*Sus*). Two samples, Sample 15: *Bos* (cattle) (18.1) and *Sus* (pig) (78.1) and Sample 135: *Bos* (cattle) (38.8) and *Sus* (pig) (60.1) can be considered as a mislabeled sample because the *Bos* (beef) genus represented a smaller percentage than the predominant *Sus* (pork) genus (Table S11).

### 3.6. Proportion Test of Two Categories (Contaminated vs. Not Contaminated) Using a Chi-Square Test

**Figure 12:**
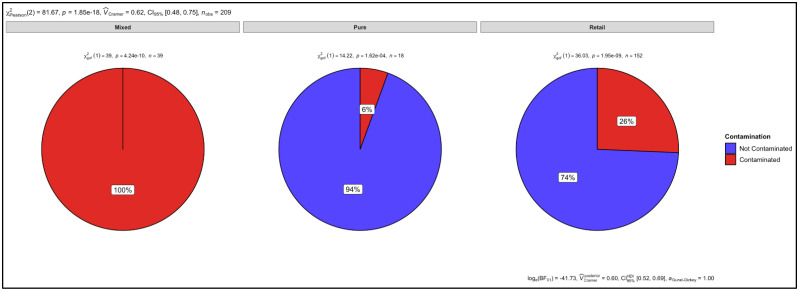
Proportional test for mixed, pure, and retail samples.

*P*-values of *p* = 1.62 × 10^−4^, *p* = 4.24 × 10^−10^ and *p* = 1.95 × 10^−9^ were determined as a result of the chi-square test for the number of contaminated versus uncontaminated pure, artificially mixed and retail samples, respectively (Figure 12). There was 6% contamination in the pure samples and there was no statistically significant level of contamination. However, there was 100% and 26% contamination in the mixed and retail samples, respectively. The *p* values therefore indicate a significant level of contamination in the artificially mixed and retail samples. The overall *p* value for all three sample groups was *p* = 1.85 × 10^−18^. Cramer’s V association was 0.62, confidence interval (CI) was set at 95% (0.48, 0.75) and the number of samples observed (n_obs_) was 209 (Figure 12).

**Figure 13:**
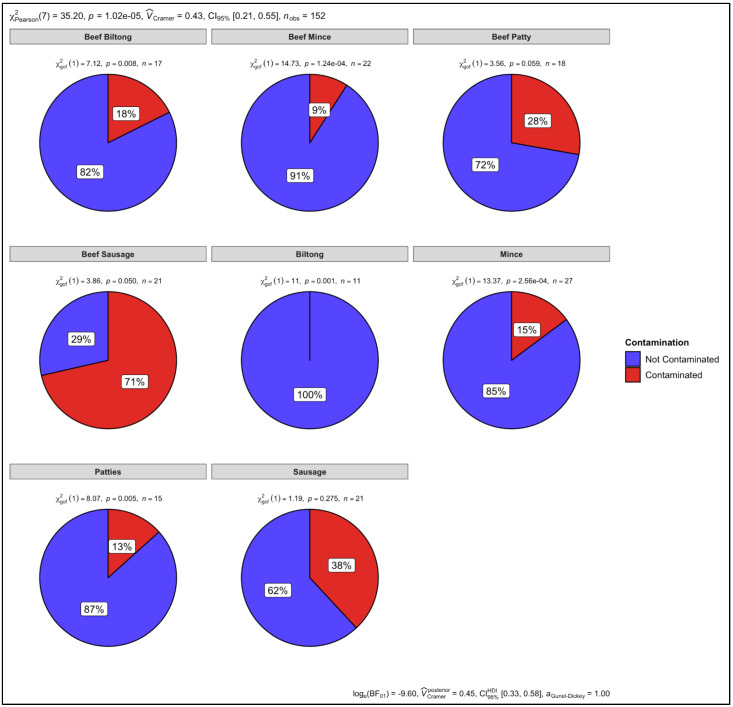
Proportional test for biltong, mince, patty, and sausage samples.

The retail samples were then statistically analysed, according to the different meat types. There was no contamination observed in the biltong samples that had not specified which species they were from and the *p*-value (*p* = 0.001) shows that there was no statistically significant level of contamination (Figure 13). There was contamination observed in the mince (15%), patties (13%), and sausage (38%) samples that had not specified which species they were from. The *p*-values for the mince (*p* = 2.56 × 10^−4^) and patty (*p* = 0.005) samples indicate that there was a statistically significant level of contamination, however, there was no statistically significant level of contamination in the sausage (*p* = 0.275) samples (Figure 13).

There was 18%, 9%, and 29% contamination in the beef biltong (*p* = 0.008), beef mince (*p* = 1.24 × 10^−4^), and beef sausage samples (*p* = 0.050), indicating a statistically significant level of observed contamination. However, regardless of finding 28% contamination in the beef patty samples, there was no statistically significant level of contamination (*p* = 0.059) (Figure 13). The overall *p* value for all the retail samples was *p =* 1.02 × 10^−5^. Cramer’s V association was 0.48, confidence interval (CI) was set at 95% (0.21, 0.55) and the number of samples observed (n_obs_) were 152 (Figure 13).

## 4. Discussion

The main aim for this work was to develop a diagnostic pipeline for species identification in meat samples, including the identification of species in artificially mixed samples from different mammalian species. The mitochondrial 16S rRNA marker used in this study has proven, in earlier studies, to have the power to detect individual species and even distinguish between closely related species [17].

A pipeline, using the mitochondrial 16S rRNA gene and NGS, was established to initially identify known meat types from pure DNA that were not mixed with any other meat types. To achieve this, pure DNA from the respective meat types was used. The overall results demonstrated the ability of our protocol to identity pure DNA that is not mixed with other meat types, as all meat types from the known pure DNA were identified and mapped to the corresponding genera. The reads obtained mapped to the corresponding genus of each pure meat sample, with a similarity of ≤98% relative abundance for all meat types and with minor deviations of less than 2% relative abundance. One ostrich sample that was contaminated with beef DNA was a result of human error in the lab.

Contamination can either be intentional or unintentional. Intentional contamination occurs when deliberately adding a cheaper material to a product for economic gain. Unintentional contamination is the mistaken introduction of something into a product. This usually occurs through cross-contamination from the use of the same equipment amongst different products [3].

In our experiment, the initial aim was to test the pipeline using 100% pure meat samples. There was no intention to assess contamination with other meat types. However, a contamination of less than 2% from other meat types was observed, which could be attributed to (i) trace amounts of other species having occupied abattoirs, butcheries, and retailers that slaughter, process, and sell multiple species’ meat and use the same equipment for their processing, or (ii) a lack of maintenance of sequence databases could affect the stringency or sensitivity of species identification pipelines if new information is not added to a given database.

Overall, the initial verification test enabled us to determine some of the sensitivity thresholds of the pipeline. A threshold of 1% (*w*/*w*) for undeclared meat species in meat products was set by the Food Safety Authority (FSA) and Department for Environment Food and Rural Affairs (Defra) in Europe, after horse and pig DNA was identified in beef products [32]. Based on our analysis, a threshold of 2% is more practical when considering cross-contamination and database-stringency factors. However, a threshold of 2% carries implications for consumers who are mindful of their diet for religious reasons. The Jewish and Muslim communities prioritize the traceability and authenticity of the meat they consume, because they only consume meat from ritually slaughtered animals in accordance with their beliefs [33]. Not declaring the meat species composition in a product violates their rights as consumers.

Having established the workflow and thresholds, the pipeline was further used to identify meat types from artificially mixed DNA at ratios of either 1:1 or 9:1. The aim of this part of the experiment was to simulate the conditions of retail market and determine whether the pipeline could identify species in mixed DNA. The 1:1 ratio simulated retailers that intentionally contaminate meat products and do not try to hide it. This type of contamination mainly occurs for economic gain, by intentionally adding a cheaper product to the primary product [1,7,29]. The pipeline demonstrated that the DNA of all meat types in the 1:1 ratio mixture were identified. However, some deviations were observed from the expected 50:50 percentages, and minute traces of species not included in some ratio mixtures. The major deviations were observed amongst the mutton: chevon, duck: ostrich and beef: kangaroo ratio mixtures. The deviation in the mutton: chevon ratio mixture may have been due to sheep and goats having similarities between their genomes, since they originate from the same family, *Bovidae,* and sub-family *Caprinae*. Previous research has demonstrated that sheep and goats evolved from the same ancestor *Rupicaprids* (goat antelopes) in the Pleistocene era [15,34]. This may have resulted in an over or underestimation in the mutton: chevon ratio mixture. Similarly, ducks and ostriches have similarities in their genomes as they are both *Aves* species. Previous comparative cytogenic work has suggested that there is a preserved sequence homology between the Z and W chromosomes in ducks and ostriches, since recombination was suppressed [35]. Furthermore, research has also demonstrated that the ostrich IgM isotype has a 66% and 63.1% sequence identity with the Cα and Cµ genes of the duck, respectively [35]. The overestimation of beef meat and underestimation of kangaroo meat in the beef: kangaroo ratio mixture may have been due to the cattle genome being sequenced more than the kangaroo genome. The sequencing of the bovine genome was initiated in 2002 [36] and has continued, with several other works since published [37,38,39,40,41]. The kangaroo genome, on the other hand, was only first sequenced in 2011 [42,43], even though the benefits of sequencing the kangaroo genome where initially discussed and published in 2003 [44]. Further research on sequencing the kangaroo genome has been published [45,46], but there is a clear indication from the number of published articles that the kangaroo genome has been sequenced less than the cattle genome. Therefore, this may have resulted in an overestimation of cattle reads in the beef: kangaroo ratio mixture. The ever-decreasing cost of sequencing, coupled with increased efforts in sequencing non-conventional livestock species such as the kangaroo, will improve on the composition and quality of databases, which will also improve on the accuracy of the methods developed to date.

The 9:1 ratio simulated retailers that also intentionally contaminate meat products but try to hide it. This type of contamination occurs in situations where retailers intentionally add the fat or trimmings of certain meat species, such as pork, to improve the sensory value of some products [31]. Similar to the results of the 1:1 ratio mixtures, the pipeline also managed to identify all the meat species whose DNA were in the 9:1 ratio, with some deviations from the expected 90:10 percentages. Descriptive statistics for the pure and artificially mixed samples were analysed. The standard deviation and variance indicated how close an observed value in a dataset is to the mean. A dataset with a smaller spread of values results in values closer to the mean, yielding smaller variance and standard deviation. In contrast, if a dataset has a wider spread of values, this results in values that are further from the mean, yielding a larger variance and standard deviation [47]. The pure samples had lower standard deviations and variances that were closer to zero, meaning the values in the dataset had a smaller range and mean value. In contrast to the pure samples, the artificially mixed samples had higher standard deviation and variance values. This was brought about by a higher range within the dataset. The higher range of values may have been a result of the under and overestimation of expected percentages in the ratio mixtures. The use of mitochondrial genes in species identification has been used due to mitochondria having a mutation rate that is 10-fold higher than that of nuclear genes, allowing for the discrimination of closely related species. Mitochondria is also abundant, with thousands of copies of DNA per cell, in comparison to nuclear genes that have single copies per cell [48]. However, the presence of several copies of mitochondrial DNA in a single cell can lead to either an underestimation (−70%) or overestimation (+160%) of species’ DNA content [6]. It has also been previously reported that there is a difference in binding efficiency of the universal primers for different species, resulting in a difference in the amplification efficiency and, therefore, leading to a large degree of error in quantitative analysis [32]. The quantitative accuracy in meat species identification can be improved through the use of genes that have a single copy, the introduction of correction factors for primer amplification efficiency, designing degenerate primers, and controlling the number of amplification cycles and the amplification conditions [32].

The analyses of retail meat samples showed that beef was the main species found in most samples, since their reads predominantly mapped to the *Bos* genus. The samples that had indicated which species they were from on their product labels predominantly mapped to the *Bos* (cattle) genus (90% and above), confirming their origination from beef, as stated on the labels. There was, however, evidence of contamination and mislabeling of the pork (*Sus*) and/or mutton (*Ovis*) meat observed in most samples, but no mention of the presence of any other species on the labelling. All the unspecified biltong samples predominantly mapped to the *Bos* genus, however, there were minor traces of te *Sus* (pig) and *Ovis* (sheep) genera of less than 2% relative abundance, hence we concluded that there was no intentional contamination in the non-specified biltong samples. The beef biltong samples showed that pork had the highest percentage of contamination, with one of the samples having as high as 36% of the reads mapping to Sus genus. The contamination seemed intentional and for economic gain, as it is not practical to mistakenly add 36% of a different meat species, especially if it is a meat type that has been previously reported to have a cheaper purchase price [1]. Bottaro et al., 2014 [49] also reported of addition of low-valued meat and fat, such as pork, to high-valued meat, such as beef, as a form of intentional meat contamination for the purposes of economic gain.

Similarly, specified and unspecified mince samples showed that the *Bos* (cattle) genus was predominant, with few samples contaminated with pork or mutton. There were a few contaminated mince samples that were contaminated with either mutton or pork. The percentages of pork found in the mince samples were between 2–3% relative abundance. These contamination percentages of may not necessarily have occurred intentionally for economic gain, since they were found at low percentages. Rather, the contamination may have been due to cross contamination from equipment not properly cleaned in operations that process multiple species [29]. Similarly, in a South African study, Tembe, Mukaratirwa, and Zishiri, 2018 [29] concluded that the contamination of processed meat products was unintentional, and that the contamination may have been due to the use of the same equipment for processing different species. Regardless of the low contamination percentages in our study, the presence of pork has negative consequences to consumers who choose not to consume pork due to health reasons [7]. The consumption of meat with undeclared allergens may cause an allergic reaction to certain consumers [7,50,51]. Previously, in the United States, allergy prevalences of 73%, 58%, and 41% to beef, pork and chicken, respectively, were reported in 57 patients suspected of being allergic to meat [52]. The patty samples were also mainly from beef meat, with samples predominantly mapping to the *Bos* (cattle) genus. Pork was the main contaminate in the unspecified patty samples. One sample (Sample 48) had 7% pork in it, possibly a case of intentionally adding pork to a patty sample to improve its sensory and oxidative properties. The addition of pork meat or lard to processed meat products has been previously reported [33], to improve the sensory properties and oxidative reactions of such processed meat products. Patty Sample 85 was intentionally mixed with two meat types, pork and beef, as 59% of its read mapped to the Sus (pig) genus and 40% mapped to the *Bos* (cattle) genus. A case like this demonstrates intentional contamination for economic gain (unless if specified on the product label), since the production costs of pork are cheaper than those of beef [1], resulting in pork being cheaper to purchase. The major contaminant of labelled beef patty products was mutton (Ovis). This was unexpected, as mutton has a higher market price than beef. One of the reasons for substituting more expensive meat such as mutton for a cheaper meat such as beef may be due to the use of unmarketable trimmings from more expensive meat types [7]. It is possible that intentional contamination with unmarketable mutton occurred in the contaminated beef patty samples, as one of the samples had as high as 34% of its reads map to the Ovis (mutton) genus.

Our results demonstrated that pork and mutton were the main species that were contaminated in the sausage samples that had reads that predominately mapped to the *Bos* (beef) genus. However, there were samples of mutton and pork sausages contaminated with beef at percentages of 2–7% relative abundance. Some authors [21,49] have indicated that the contamination of beef, in some meat products, may be from the addition of non-fat powdered milk to increase the overall yield and taste of the product. This may have been the case with the mutton and pork samples contaminated with beef. There was one sample (Sample 15) that was intentionally mixed with three meat types, beef, pork, and mutton, as the sample had 37%, 38%, and 23% of reads map to the *Bos* (cattle), *Sus* (pig), and *Ovis* (sheep) genus, respectively. This may have been a case of contamination for economic gain, because mutton and beef have a higher market price than pork [29]. The overall results of the specified and unspecified meat products indicated that pork was the main contaminate. Surowiec et al., 2011 [53] previously reported on undeclared pork and chicken in processed meat products such as burger patties and sausages and suggested that it could be from mechanically recovered meat (MRM), usually produced from pork and chicken carcasses. According to Surowiec et al., 2011 [53], the addition of MRM, which is normally found in a paste-like form, represents a source of cheap protein in processed meat products, such as deli meats, burger patties, and sausages. This practice is, however, illegal in most countries, including South Africa [1]. Furthermore, failure to declare the presence of other meat species in ingredients lists betrays consumer rights and has negative implications for consumers allergic to such contaminants and consumers whose religions observe dietary restrictions [7].

Similarly, in a South African study [1], undeclared pork and mutton were found in minced meat, burger patties, and raw sausages labelled as beef, pork meat was the main undeclared meat type found in these meat samples. In another South African study on meat species’ substitution, undeclared beef, pork, and lamb were found in commercially labelled wildlife meat products [2]. More recently, another South African study revealed the presence of meat contamination in the province of KwaZulu-Natal [29]. A high proportion of beef and mutton products were contaminated with pork and chicken. Undeclared species in the above-mentioned studies and in ours reflect that there is still a presence of meat adulteration in the South African meat market. Judging from the results we have observed from the retail samples, there seems to be intentional contamination for economic gain. There is need to improve product labelling so as to indicate every species within a meat product so that consumers can make informed decisions. Some major retailers in South Africa, such as Food Lover’s Market, Pick and Pay, and Checkers, now mention the presence of multi-species on their meat product’s labels. For example, a sausage sample, today, might be labelled as 70% beef, 20% pork, and 10% Water. This type of clarity in labelling assists consumers who prefer to avoid certain species for allergenic, religious, or ethical reasons.

Statistics indicate that beef has the highest gross value as compared with other meat species produced in South Africa, with an average of R 23.5 billion per annum [54]. There was a slight decrease in cattle production between 2017–2018, due to farmers in South Africa not having enough cattle to slaughter. This led to an increase in beef market prices, as herds were replenished, which in turn decreased the consumption of beef and beef products. Consumers opted for cheaper alternatives, such as chicken and pork [54]. The gross production of mutton in South Africa is an average of R 4.57 billion per annum [55]. There was, however, a decline in sheep production from 2017 due to stock theft, which led to an increase in demand and subsequent shortages in the supply of mutton [55]. This shortage in supply, coupled with high production costs, has led to high market prices of mutton in the South African meat market. Beef is more readily available, hence it is the main species processed into value-added products in South Africa. However, high production costs result in a higher purchasing price for beef. Mutton also has a higher purchasing price than the other meat types, mainly because it has higher production costs and is not readily available on the market. Chicken and pork have lower production costs in comparison with beef and mutton and this has led to them having a lower purchasing price and, therefore, being more frequently fraudulently added to higher-value products labelled as beef or mutton, for economic gain.

## 5. Conclusions

In conclusion, the current paper presents a universal diagnostic molecular method for the identification of meat species. The method used the mitochondrial 16S rRNA gene, which has demonstrated its variability from the results of the phylogenetic analysis in our previous study. The verification experiment identified all species present in the known DNA mixtures, proving the accuracy of the pipeline in the species identification in the processed meat samples that were collected. Meat suppliers can possibly implement the current method to authenticate their products, and the food industry may also use this method to reveal any form of mislabeling that may be present within meat productsi.

## Figures and Tables

**Table 1:** Ratios and species of artificial DNA mixtures.

Mixture Ratio	Species 1: Species 2
1:1	pork ‖ beef, mutton ‖ chevon, chicken ‖ turkey, ostrich ‖ duck and beef ‖ kangaroo
9:1	pork ‖ beef, pork ‖ beef, mutton ‖ chevon, chevon ‖ mutton, chicken ‖ duck, duck ‖ chicken, ostrich ‖ duck and duck ‖ ostrich

**Table 2:** The oligodeoxynucleotide sequences of the universal primers for 16S rRNA gene amplification designed by Tillmar et al., 2013 [17] (the letters in small case are Nextera adapter tails).

16S Forward	5′tcgtcggcagcgtcagatgtgtataagagacagGACGAGAAGACCCTATTGGAGC 3′
16S Reverse	5′gtctcgtgggctcggagatgtgtataagagacagTCCGAGGTCRCCCCAACC 3′

**Table 3:** Descriptive statistics of artificially mixed and pure samples.

		Artificially Mixed % Deviation			
**Min.**	**Max.**	**Median**	**Mean**	**Standard Deviation**	**Variance**
1.0	24.59	9.08	13.76	6.98	48.66
		**Pure % Deviation**			
**Min.**	**Max.**	**Median**	**Mean**	**Standard Deviation**	**Variance**
0.30	3.49	1.184	1.56	0.716	0.513

## Data Availability

Data is contained within the article or Supplementary Material.

## Supplementary Materials

The following are available online at https://www.mdpi.com/article/10.3390/ani12040416/s1, Tables S1–S11: Average fold values for pure meat, mixed ratios, and processed meat samples.
