# Supplementary material for: A 16S Next Generation Sequencing Based Molecular and Bioinformatics Pipeline to Identify Processed Meat Products Contamination and Mislabelling
Source: Animals (Basel). 2022 Feb 10;12(4):416. doi: 10.3390/ani12040416 (PMC8868451; doi:10.3390/ani12040416)
Supplement: Supplementary file 1 [file animals-12-00416-s001.zip › animals-1356191-supplementary.pdf]

Supplementary Tables

Table S1. Percentage average fold of pure DNA from known meat types.

Known Meat type (100%)	Genus (% Of Average Fold)	Percentage deviation
Beef	*Bos* (Cattle) (99), *Bubalus* (Buffalo) (0,4), *Struthio* (Ostrich) (0,2) & *Rupicapra* (Goat antelope) (0,4)	1
Mutton	*Ovis* (Sheep) (99,1), *Bos* (Cattle) (0,1), *Rupicapra* (Goat Antelope) (0,6), *Naemorhedus* (Goral) (0,1) & *Anas* (Duck) (0,1)	0.9
Pork	*Sus* (Pig) (99,7), *Ovis* (Sheep) (0,2), & *Bos* (Cattle) (0,1)	0.3
Chevon	*Capra* (Goat) (98,7), *Ovis* (Sheep) (0,2), *Budorcas* (Gnu Goat), *Bos* (Cattle) (0,4), *Rupicapra* (Goat Antelope) (0,2), & *Sus* (Pig) (0,1)	1.3
Chicken	*Gallus* (Chicken) (98,5), *Bos* (Beef) (0,3), *Sus* (Pig) (0,2), *Capra* (Goat) (0,6), & *Ovis* (Sheep) (0,3)	1.4
Duck	*Anas* (Duck) (98,5), *Capra* (Goat) (0,1), *Ovis* (Sheep) (0,5), *Gallus* (Chicken) (0,2), *Bos* (Beef) (0,1), *Meleagris* (Turkey) (0,3), & *Sus* (Pig) (0,2)	1.5
Turkey	*Meleagris* (Turkey) (99,1), *Sus* (Pig) (0,1), *Anas* (Duck) (0,2), *Capra* (Goat) (0,2), *Ovis* (Sheep) (0,1), *Gallus* (Chicken) (0,1), & *Bos* (Beef) (0,1)	0.9
Ostrich	*Struthio* (Ostrich) (99,3), *Bos* (Beef) (0,3), *Meleagris* (Turkey) (0,1), *Sus* (Pig) (0,1), *Anas* (Duck) (0,1), & *Ovis* (Sheep) (0,1)	0.7
Kangaroo	*Macropus* (Kangaroo) (99,4), *Capra* (Goat) (0,2), *Struthio* (Ostrich) (0,2), *Bos* (Cattle) (0,1), & *Anas* (Duck) (0,1)	0.6

Table S2. Percentage of average fold of pure DNA from two known meat types artificially mixed at a ratio of 1:1

Mixture Ratio	Meat Type (%)	Genus (% Of Average fold)	Percentage Difference with Respect to Input Ratio
1:1	Pork (50%)<br>Beef (50%)	*Sus* (Pig) (51,5)<br>*Bos* (Cattle) (48,1)<br>*Ovis* (Sheep) (0,1)<br>*Bubalus* (Buffalo) (0,1)<br>*Rupicapra* (Goat Antelope) (0,1)	1,5<br>-1,9
1:1	Chevon (50%)<br>Mutton (50%)	*Capra* (Goat) (35,7)<br>*Ovis* (Sheep) (63,4)<br>*Budorcas* (Gnu Goat) (0,1)<br>*Bos* (Cattle) (0,4)<br>*Rupicapra* (Goat Antelope) (0,3)<br>*Sus* (Pig) (0,1)	-14,3<br>13,4
1:1	Chicken (50%)<br>Turkey (50%)	*Gallus* (Chicken) (45,3)<br>*Meleagris* (Turkey) (50,8)<br>*Bos* (Cattle) (0,2)<br>*Sus* (Pig) (0,1)	-4,7<br>0,8

		Capra (Goat) (0,3)	
		Ovis (Sheep) (0,2)	
1:1	Duck (50%)	*Anas* (Duck) (69,2)	19,2
	Ostrich (50%)	*Struthio* (Ostrich) (29,9)	-20,1
		Sus (Pig) (0,2)	
		Capra (Goat) (0,1)	
		Ovis (Sheep) (0,2)	
		Gallus (Chicken) (0,1)	
		Bos (Cattle) (0,3)	
1:1	Beef (50%)	*Bos* (Cattle) (73,9)	23,9
	Kangaroo (50%)	*Macropus* (Kangaroo) (25,1)	24,9
		Bubalus (Buffalo) (0,2)	
		Gallus (Chicken) (0,1)	
		Rupicapra (Goat Antelope) (0,2)	
		Sus (Pig) (0,2)	
		Anas (Duck) (0,1)	

Table S3. Percentage average folds of pure DNA from known meat samples artificially mixed at a ratio of 9:1.

Mixture Ratio	Meat Type (%)	Genus (% Of Average Fold)	% Difference with Respect to Input Ratio
9:1	Pork (90%)	*Sus* (Pig) (91,3)	1,3
	Beef (10%)	*Bos* (Cattle) (8,5)	-1,5
		Ovis (Sheep) (0,1)	
9:1	Beef (90%)	*Bos* (Cattle) (92,7)	2,7
	Pork (10%)	*Sus* (Pig) (6,6)	-3,4
		Bubalus (Buffalo) (0,3)	
		Rupicapra (Goat Antelope) (0,3)	
9:1	Chevon (90%)	*Capra* (Goat) (81,5)	-8,5
	Mutton (10%)	*Ovis* (Sheep) (17,4)	7,4
		Budorcas (Gnu Goat) (0,3)	
		Bos (Cattle) (0,1)	
		Rupicapra (Goat Antelope) (0,2)	
	Mutton (90%)	*Ovis* (Sheep) (92,7)	2,7
	Chevon (10%)	*Capra* (Goat) (6,4)	-3,6
		Rupicapra (Goat Antelope) (0,5)	
		Naemorhedus (Goral) (0,1)	
9:1	Chicken (90%)	*Gallus* (Chicken) (82,1)	-7,9
	Duck (10%)	*Anas* (Duck) (17,5)	7,5
		Capra (Goat) (0,1)	

		Ovis (Sheep) (0,1)<br>*Sus* (Pig) (0,1)<br>*Bos* (Cattle) (0,1)	
9:1	Duck (90%)<br>Chicken (10%)	*Anas* (Duck) (96,3)<br>*Gallus* (Chicken) (3,4)<br>*Sus* (Pig) (0,1)	6,3<br>-3,6
9:1	Ostrich (90%)<br>Duck (10%)	*Struthio* (Ostrich) (81,2)<br>*Anas* (Duck) (18,5)<br>*Bos* (Cattle) (0,1)	-8,8<br>8,5
9:1	Duck (90%)<br>Ostrich (10%)	*Anas* (Duck) (86,4)<br>*Struthio* (Ostrich) (13,2)<br>*Ovis* (Sheep) (0,1)<br>*Sus* (Pig) (0,1)<br>*Bos* (Cattle) (0,1)	-3,6<br>3,2

Table S4. Percentage average fold of DNA from retail biltong for which the meat types were not indicated on product labels (N=11).

Sample Number	Genus (% Of Average fold)
S19	*Bos* (Cattle) (98,1), *Bubalus* (Buffalo) (0,6), *Rupicapra* (Goat Antelope) (0,8), *Redunca* (Reedbuck) (0,2) and *Bison* (Bison) (0,1)
S35	*Bos* (Cattle) (99,7), & *Bubalus* (Buffalo) (0,1)
S3	*Bos* (Cattle) (98,3), *Bubalus* (Buffalo) (0,7), *Rupicapra* (Goat Antelope) (0,7), *Redunca* (Reedbuck) (0,2) and *Bison* (Bison) (0,1)
S45	*Bos* (Cattle) (99,4), *Bubalus* (Buffalo) (0,2), *Rupicapra* (Goat Antelope) (0,1), *Sus* (Pig) (0,1) and *Redunca* (Reedbuck) (0,1)
S54	*Bos* (Cattle) (98,2), *Bubalus* (Buffalo) (0,7), *Redunca* (Reedbuck) (0,3), *Sus* (Pig) (0,2), *Hemitragus* (Tahr) (0,2), *Capricornis* (Serow) (0,1) and *Bison* (Bison) (0,1)
S56	*Bos* (Cattle) (99,4), *Bubalus* (Buffalo), (0,2), *Rupicapra* (Goat Antelope) (0,1), *Sus* (Pig) (0,1) and *Redunca* (Reedbuck) (0,1)
S62	*Bos* (Cattle) (98,2), *Rupicapra* (Goat Antelope) (0,6), *Bubalus* (Buffalo) (0,5), *Redunca* (Reedbuck) (0,2), *Capricornis* (Serow) (0,1), *Bison* (Bison) (0,1), and *Hemitragus* (Tahr) (0,1)
S67	*Bos* (cattle) (98,7), *Bubalus* (Buffalo) (0,5), *Rupicapra* (Goat Antelope) (0,4), *Redunca* (Reedbuck) (0,2) and *Bison* (Bison) (0,1)
S69	*Bos* (Cattle) (99,4), *Bubalus* (Buffalo) (0,2), *Bison* (Bison) (0,1), *Rupicapra* (Goat Antelope) (0,2) and *Redunca* (Reedbuck) (0,1),
S92	*Bos* (Cattle) (xx) (98,4), *Rupicapra* (Goat Antelope) (0,7), *Bubalus* (Buffalo) (0,5) and *Redunca* (Reedbuck) (0,2)
S09	*Bos* (Cattle) (99,2), *Bubalus* (Buffalo) (0,3), *Rupicapra* (Goat Antelope) (0,2) *Sus* (Pig) (0,1) and *Redunca* (Reedbuck) (0,1)

Table S5. Percentage average fold of DNA from retail meat products labelled as beef biltong (N=17)

Sample Number	Genus (% Of Average Fold)
S©	*Bos* (Cattle) (**93,6**), *Ovis* (Sheep) (4,9), *Sus* (Pig) (0,8), *Bubalus* (Buffalo) (0,2) *Bison* (Bison) (0,1), *Rupicapra* (Goat Antelope) (0,1) and *Redunca* (Reedbuck) (0,1)
112	*Bos* (Cattle) (98,4), *Bubalus* (Buffalo) (0,6), *Rupicapra* (Goat Antelope) (0,6), *Redunca* (Reedbuck) (0,2), *Bison* (Bison) (0,1) and *Ovis* (Sheep) (0,1)

**117**©	*Bos* (Cattle) (**97,9**), *Bubalus* (Buffalo) (0,7), *Hemitragus* (Tahr) (0,3), *Capricornis* (Serow) (0,2), *Redunca* (Reedbuck) (0,2), *Moschus* (Musk-deer) (0,1), *Sus* (Pig) (0,1), *Bison* (Bison) (0,1) and *Rupicapra* (Goat Antelope) (0,1)
137	*Bos* (Cattle) (99,0), *Bubalus* (Buffalo) (0,4), *Rupicapra* (Goat Antelope) (0,2), *Redunca* (Reedbuck) (0,2), *Sus* (Pig) (0,1) and *Bison* (Bison) (0,1)
138	*Bos* (Cattle) (99,0), *Bubalus* (Buffalo) (0,4), *Rupicapra* (Goat Antelope) (0,3), *Redunca* (Reedbuck) (0,2), *Sus* (Pig) (0,1) and *Bison* (Bison) (0,1)
142	*Bos* (Cattle) (99,5), *Bubalus* (Buffalo) (0,2), *Bison* (Bison) (0,1) and *Redunca* (Reedbuck) (0,1)
147	*Bos* (Cattle) (99,2), *Sus* (Pig) (0,2), *Bubalus* (Buffalo) (0,2), *Ovis* (Sheep) (0,1), *Rupicapra* (Goat Antelope) (0,1) and *Redunca* (Reedbuck) (0,1)
148	*Bos* (Cattle) (99,0), *Bubalus* (Buffalo) (0,5), *Redunca* (Reedbuck) (0,2), *Sus* (Pig) (0,1) and *Hemitragus* (Tahr) (0,1)
149	*Bos* (Cattle) (99,4), *Bubalus* (Buffalo) (0,3), *Ovis* (Sheep) (0,1) and *Redunca* (Reedbuck) (0,1)
15	*Bos* (Cattle) (99,4), *Sus* (Pig) (0,1), *Bubalus* (Buffalo) (0,1), *Rupicapra* (Goat Antelope) (0,1) and *Redunca* (Reedbuck) (0,1)
153	*Bos* (Cattle) (99,6), *Sus* (Pig) (0,2) and *Bubalus* (Buffalo) (0,1)
**16**©	*Bos* (Cattle) (**57,5**), *Sus* (Pig) (37,4), *Ovis* (Sheep) (4,6), *Bubalus* (Buffalo) (0,1) and *Rupicapra* (Goat Antelope) (0,1)
166	*Bos* (Cattle) (99,3), *Bubalus* (Buffalo) (0,2), *Rupicapra* (Goat Antelope) (0,2) and *Redunca* (Reedbuck) (0,1)
168	*Bos* (Cattle) (98,6), *Bubalus* (Buffalo) (0,6), *Rupicapra* (Goat Antelope) (0,4), *Redunca* (Reedbuck) (0,2) and *Bison* (Bison) (0,1)
17	*Bos* (Cattle) (99,2), *Bubalus* (Buffalo) (0,4), *Rupicapra* (Goat Antelope) (0,2) and *Redunca* (Reedbuck) (0,1)
175	*Bos* (Cattle) (99,5), *Bubalus* (Buffalo) (0,3), *Sus* (Pig) (0,1) and *Redunca* (Reedbuck) (0,1)
19	*Bos* (Cattle) (99,1), *Bubalus* (Buffalo) (0,4), *Rupicapra* (Goat Antelope) (0,2), *Bison* (Bison) (0,1) and *Redunca* (Reedbuck) (0,1)

Table S6. Percentage average folds of DNA from retail minced meat for which the meat types were not indicated on product labels (N=27).

Sample Number	Genus (% Of Average fold)
S	*Bos* (Cattle) (99,7), *Bubalus* (Buffalo) (0,1) and *Rupicapra* (Goat Antelope) (0,1)
16	*Bos* (Cattle) (99,0), *Sus* (Pig) (0,6), *Bubalus* (Buffalo) (0,1) and *Rupicapra* (Goat Antelope) (0,1)
2	*Bos* (Cattle) (99,4), *Bubalus* (Buffalo) (0,3), *Bison* (Bison) (0,1) and *Redunca* (Reedbuck) (0,1)
23	*Bos* (Cattle) (99,4), *Bubalus* (Buffalo) (0,3), *Bison* (Bison) (0,1) and *Redunca* (Reedbuck) (0,1)
3	*Bos* (Cattle) (99,2), *Rupicapra* (Goat Antelope) (0,2), *Sus* (Pig) (0,1), *Ovis* (Sheep) (0,1) and *Redunca* (Reedbuck) (0,1)
33	*Bos* (Cattle) (99,5), *Bubalus* (Buffalo) (0,2), *Rupicapra* (Goat Antelope) (0,2) and *Redunca* (Reedbuck) (0,1)
**34**©	*Bos* (Cattle) (**97,7**), *Ovis* (Sheep) (1,8), *Bubalus* (Buffalo) (0,2), *Rupicapra* (Goat Antelope) (0,1) and *Redunca* (Reedbuck) (0,1)
39	*Bos* (Cattle) (99,3), *Rupicapra* (0,3), *Bubalus* (0,2) and *Sus* (Pig) (0,1)
**4**©	*Sus* (Pig) (**97,7**) and *Bos* (Cattle) (2,2)
5	*Bos* (Cattle) (99,7) and *Bubalus* (Buffalo) (0,1)
55	*Bos* (Cattle) (99,4), *Bubalus* (Buffalo) (0,2), *Rupicapra* (Goat Antelope) (0,1) and *Redunca* (Reedbuck) (0,1)
58	*Bos* (Cattle) (98,2), *Bubalus* (Buffalo) (0,8), *Rupicapra* (Goat Antelope) (0,5) and *Redunca* (Reedbuck) (0,3)
59	*Bos* (Cattle) (99,7) and *Bubalus* (Buffalo) (0,1)

6	*Bos* (Cattle) (98,8), *Sus* (Pig) (0,5), *Bubalus* (Buffalo) (0,2), *Rupicapra* (Goat Antelope) (0,3), *Ovis* (Sheep) (0,1) and *Redunca* (Reedbuck) (0,1)
63	*Bos* (Cattle) (99,6), *Bubalus* (Buffalo) (0,1) and *Rupicapra* (Goat Antelope) (0,1)
**65**©	*Bos* (Cattle) (**83,2**), *Ovis* (Sheep) (16,4), *Rupicapra* (0,2) and *Bubalus* (Buffalo) (0,1)
66	*Bos* (Cattle) (99,3), *Bubalus* (Buffalo) (0,2), *Rupicapra* (Goat Antelope) (0,1), *Ovis* (Sheep) (0,1) and *Redunca* (Reedbuck) (0,1)
74	*Bos* (Cattle) (98,3), *Sus* (Pig) (1,3), *Bubalus* (Buffalo) (0,2), *Rupicapra* (Goat Antelope) (0,1) and *Redunca* (Reedbuck) (0,1)
77	*Bos* (Cattle) (99,0), *Bubalus* (Buffalo) (0,3), *Rupicapra* (Goat Antelope) (0,3), *Bison* (Bison) (0,1) and *Redunca* (Reedbuck) (0,1)
**78**©	*Bos* (Cattle) (**95,3**), *Sus* (Pig) (3,8), *Bubalus* (Buffalo) (0,2), (Goat Antelope) (0,3), *Bison* (Bison) (0,1), *Rupicapra* and *Redunca* (Reedbuck) (0,1)
8	*Bos* (Cattle) (99,6), *Bubalus* (Buffalo) (0,1) and *Rupicapra* (Goat Antelope) (0,1)
89	*Bos* (Cattle) (98,8*)*, *Ovis* (Sheep) (0,4), *Sus* (Pig) (0,2), *Bubalus* (Buffalo) (0,2), *Rupicapra* (Goat Antelope) (0,2) and *Redunca* (Reedbuck) (0,1)
9	*Bos* (Cattle) (99,4), *Bubalus* (Buffalo) (0,2), *Rupicapra* (Goat Antelope) (0,2), *Sus* (Pig) (0,1) and *Redunca* (Reedbuck) (0,1)
93	*Bos* (Cattle) (99,5), *Bubalus* (Buffalo) (0,2), *Rupicapra* (Goat Antelope) (0,1) and *Redunca* (Reedbuck) (0,1)
94	*Bos* (Cattle) (99,8) and *Bubalus* (Buffalo) (0,1)
95	*Bos* (Cattle) (99,3), *Bubalus* (Buffalo) (0,2), *Bison* (Bison) (0,1), *Rupicapra* (Goat Antelope) (0,2) and *Redunca* (Reedbuck) (0,1)
97	*Bos* (Cattle) (99,5), *Bubalus* (Buffalo) (0,1), *Rupicapra* (Goat Antelope) (0,2), *Sus* (Pig) (0,1) and *Redunca* (Reedbuck) (0,1)

Table S7. Percentage average fold of DNA from retail meat products labelled as beef mince.

Sample Number	Genus (% Of Average Fold)
S	*Bos* (Cattle) (99,5), *Bubalus* (Buffalo) (0,2), *Rupicapra* (Goat Antelope) (0,1) and *Redunca* Reedbuck) (0,1)
109	*Bos* (Cattle) (98,9), *Bubalus* (Buffalo) (0,7), *Redunca* (Reedbuck) (0,3) and *Bison* (Bison) (0,1)
12	*Bos* (Cattle) (99,3), *Bubalus* (Buffalo) (0,3), *Rupicapra* (Goat Antelope) (0,2) and *Redunca* (Reedbuck) (0,1)
123	*Bos* (Cattle) (99,3), *Bubalus* (Buffalo) (0,2), *Sus* (Pig) (0,2), *Ovis* (Sheep) (0,1), *Rupicapra* (Goat Antelope) (0,1) and *Redunca* (Reedbuck) (0,1)
128	*Bos* (Cattle) (99,2), *Bubalus* (Buffalo) (0,3), *Rupicapra* (Goat Antelope) (0,2) and *Redunca* (Reedbuck) (0,1)
13	*Bos* (Cattle) (99,2), *Bubalus* (Buffalo) (0,2), *Sus* (Pig) (0,1), *Bison* (Bison) (0,1) and *Rupicapra* (Goat Antelope) (0,1)
132	*Bos* (Cattle) (99,6), *Sus* (Pig) (0,1), *Bubalus* (Buffalo) (0,1), *Rupicapra* (Goat Antelope) (0,1) and *Redunca* (Reedbuck) (0,1)
133	*Bos* (Cattle) (99,1), *Bubalus* (Buffalo) (0,2), *Sus* (Pig) (0,2), *Rupicapra* (Goat Antelope) (0,1), *Bison* (Bison) (0,1), *Redunca* (Reedbuck) (0,1)
139	*Bos* (Cattle) (99,4), *Bubalus* (Buffalo) (0,2) *Sus* (Pig) (0,1), *Rupicapra* (Goat Antelope) (0,1) and *Redunca* (Reedbuck) (0,1)
14	*Bos* (Cattle) (99,0), *Sus*0,3 *Bubalus*0,3 *Rupicapra*0,2 *Redunca*0,1
152	*Bos* (Cattle) (99,2), *Bubalus* (Buffalo) (0,3), *Rupicapra* (Goat Antelope) (0,2), *Sus* (Pig) (0,1) and *Redunca* (Reedbuck) (0,1)
154	*Bos* (Cattle) (99,5), *Bubalus* (Buffalo) (0,1) and *Rupicapra* (Goat Antelope) (0,1)
158	*Bos* (Cattle) (98,9), *Ovis* (Sheep) (0,4), *Sus* (Pig) (0,2), *Homo* (Human) (0,1), *Bubalus* (Buffalo) (0,1) and *Redunca* (Reedbuck) (0,1)
162	*Bos* (Cattle) (99,1), *Bubalus* (Buffalo) (0,3), *Ovis* (Sheep) (0,2), *Bison* (Bison) (0,1), *Rupicapra* (Goat Antelope) (0,1) *Redunca* (Reedbuck) (0,1)

163	*Bos* (Cattle) (99,1), *Bubalus* (Buffalo) (0,3), *Ovis* (Sheep) (0,2), *Bison* (Bison) (0,1), *Rupicapra* (Goat Antelope) (0,1) and *Redunca* (Reedbuck) (0,1)
164	*Bos* (Cattle) (99,5), *Sus* (Pig) (0,3), *Bubalus* (Buffalo) (0,1) and *Rupicapra* (Goat Antelope) (0,1)
167	*Bos* (Cattle) (98,5), *Ovis* (Sheep) (0,6), *Bubalus* (Buffalo) (0,3), *Rupicapra* (Goat Antelope) (0,3) and *Redunca* (Reedbuck) (0,1)
**17©**	*Bos* (Cattle) (**97,0**), *Ovis* (Sheep) (2,0), *Bubalus* (Buffalo) (0,3), *Rupicapra* (Goat Antelope) (0,3), *Sus* (Pig) (0,1), *Bison* (Bison) (0,1) and *Redunca* (Reedbuck) (0,1)
173	*Bos* (Cattle) (99,4), *Bubalus* (Buffalo) (0,3), *Rupicapra* (Goat Antelope) (0,1) and *Redunca* (Reedbuck) (0,1)
18	*Bos* (Cattle) (98,3), *Ovis* (Sheep) (0,7), *Bubalus* (Buffalo) (0,3), *Sus* (Pig) (0,3), *Rupicapra* (Goat Antelope) (0,3) and *Redunca* Reedbuck (0,1)
**183©**	*Bos* (Cattle) (**93,3**), *Sus* (Pig) (6,1), *Bubalus* (Buffalo) (0,2), *Rupicapra* (Goat Antelope) (0,2) and *Redunca* (Reedbuck) (0,1)
99	*Bos* (Cattle) (99,1), *Homo* (Human) (0,5) and *Struthio* (Ostrich) (0,2)

Table S8. Percentage average fold of DNA from retail patties for which the meat types were not indicated on product labels (N=15).

**Sample Number**	**Genus (% Of Average Fold)**
S	*Bos* (Cattle) (99,3), *Sus* (Pig) (0,2), *Bubalus* (Buffalo) (0,2), *Rupicapra* (Goat Antelope) (0,2) and *Redunca* (Reedbuck) (0,1)
12	*Bos* (Cattle) (99,4), *Bubalus* (Buffalo) (0,2), *Sus* (Pig) (0,1), *Ovis* (Sheep) (0,1) and Redunca (Reedbuck) (0,1)
15	*Bos* (Cattle) (99,1), *Ovis* (Sheep), (0,4), *Bubalus* (Buffalo) (0,2), *Rupicapra* (Goat Antelope) (0,1) and *Redunca* (Reedbuck) (0,1)
2	*Bos* (Cattle) (99,7), *Bubalus* (Buffalo) (0,1) *Rupicapra* (Goat Antelope) (0,1) and, *Redunca* (Reedbuck) (0,1)
22	*Bos* (Cattle) (99,7), *Bubalus* (Buffalo) (0,1) and *Rupicapra* (Goat Antelope) (0,1)
32	*Bos* (Cattle) (99,6), *Bubalus* (Buffalo) (0,2), *Rupicapra* (0,1) and *Redunca* (Reedbuck) (0,1)
44	*Bos* (Cattle) (99,5), *Bubalus* (Buffalo) (0,2) *Rupicapra* (Goat Antelope) (0, 2) and *Sus* (Pig) (0,1)
46	*Bos* (Cattle) (99,1), *Bubalus* (Buffalo) (0,3), *Rupicapra* (Goat Antelope) (0,3), *Sus* (Pig) (0,1) and *Redunca* (Reedbuck) (0,1).
47	*Bos* (Cattle) (99,7) and Bubalus (Buffalo) (0,1)
**48©**	*Bos* (Cattle) (**91,8**), *Sus* (Pig) (7,7), *Bubalus* (Buffalo) (0,1) *Bison* (Bison) (0,1), *Rupicapra* (Goat Antelope) (0,1) *Redunca* (Reedbuck) (0,1)
57	*Bos* (Cattle) (98,9), *Bubalus* (Buffalo) (0,4), *Rupicapra* (Goat Antelope) (0,4) and *Redunca* (Reedbuck) (0,1)
7	*Bos* (Cattle) (99,7), *Bubalus* (Buffalo) (0,1) and *Rupicapra* (Goat Antelope) (0,1)
73	*Bos* (Cattle) (99,2), *Bubalus* (Buffalo) (0,2) *Rupicapra* (Goat Antelope), (0,2) and *Redunca* (Reedbuck) (0,1)
8	*Bos* (Cattle) (99,6), *Sus* (Pig) (0,1) *Bubalus* (Buffalo) (0,1) and *Rupicapra* (Goat Antelope) (0,1)
**84©**	*Sus* (Pig) (**59,6**), *Bos* (Cattle) (40,0), *Bubalus* (Buffalo) (0,1), *Connochaetes* (Wildebeest) (0,1) and *Rupicapra* (Goat Antelope) (0,1)

Table S9. Percentage average folds of DNA from retail meat products labelled as beef patty (n=18)

**Sample Number**	**Genus (% Of Average Fold)**
102	*Bos* (Cattle) (99,4), *Bubalus* (Buffalo) (0,2), *Ovis* (Sheep) (0,1), *Rupicapra* (Goat Antelope) (0,1) and *Redunca* (Reedbuck) (0,1)
**113©**	*Bos* (Cattle) (**93,4**), *Ovis* (Sheep) (5,6), *Sus* (Pig) (0,1), *Bubalus* (Buffalo) (0,1) and *Rupicapra* (Goat Antelope) (0,1)

115	*Bos* (Cattle) (99,1), *Bubalus* (Buffalo) (0,3), *Ovis* (Sheep) (0,2), *Bison* (Bison) (0,1), *Rupicapra* (Goat Antelope) (0,1) and *Redunca* (Reedbuck) (0,1)
**112**©	*Bos* (Cattle) (**81,7**), *Ovis* (Sheep) (17,7) *Rupicapra* (Goat Antelope) (0,2), *Bison* (Bison) (0,1) and *Bubalus* (Buffalo) (0,1)
12	*Bos* (Cattle) (98,2), *Ovis* (Sheep) (1,2), *Rupicapra* (Goat Antelope) (0,2), *Bubalus* (Buffalo) (0,1) and *Bison* (Bison) (0,1)
**122**©	*Bos* (Cattle) (**64,5**), *Ovis* (Sheep) (34,3), *Rupicapra* (Goat Antelope) (0,7) and *Bubalus* (Buffalo) (0,1)
124	*Bos* (Cattle) (99,2), *Bubalus* (Buffalo) (0,3), *Rupicapra* (Goat Antelope) (0,2) and *Redunca* (Reedbuck) (0,1)
125	*Bos* (Cattle) (98,7), *Ovis* (Sheep) (0,9), *Bubalus* (Buffalo) (0,2), *Rupicapra* (Goat Antelope) (0,1) and *Redunca* (Reedbuck) (0,1)
13	*Bos* (Cattle) (99,5), *Sus* (Pig) (0,2) and *Bubalus* (Buffalo) (0,1)
**136**©	*Bos* (Cattle) (**94,9**), *Sus* (Pig) (4,4), *Bubalus* (Buffalo) (0,2) and *Redunca* (Rreedbuck) (0,1)
14	*Bos* (Cattle) (99,1), *Sus* (Pig) (0,2), *Bubalus* (Buffalo) (0,2), *Rupicapra* (Goat Antelope) (0,1) and *Redunca* (Reedbuck) (0,1)
146	*Bos* (Cattle) (99,5), *Sus* (Pig) (0,1), *Homo* (Huma) (0,1) and *Ovis* (Sheep) (0,1)
169	*Bos* (Cattle) (99,3), *Bubalus* (Buffalo) (0,3), *Rupicapra* (Goat Antelope) (0,1) and *Redunca* (Reedbuck) (0,1)
172	*Bos* (Cattle) (99,0), *Bubalus* (Buffalo) (0,4), *Rupicapra* (Goat Antelope) (0,2), *Redunca* (Reedbuck) (0,2) and *Bison* (Bison) (0,1)
**179**©	*Bos* (Cattle) (**93,4**), *Ovis* (Sheep) (6,2), *Bubalus* (Buffalo) (0,1) *Rupicapra* (Goat Antelope) (0,1)
18	*Bos* (Cattle) (99,3), *Bubalus* (Buffalo) (0,3), *Rupicapra* (Goat Antelope) (0,2) and *Redunca* (Reedbuck) (0,1)
182	*Bos* (Cattle) (98,7), *Homo* (Human) (0,6), *Mus* (Rat) (0,3), *Sus* (Pig) (0,1), *Bubalus* (Buffalo) (0,1) and *Redunca* (Reedbuck) (0,1)
185	*Bos* (Cattle) (98,7), *Bubalus* (Buffalo) (0,4), *Rupicapra* (Goat Antelope) (0,4), *Redunca* (Reedbuck) (0,2) and *Ovis* (Sheep) (0,1)

Table S10. Percentage average folds of DNA from retail sausages for which the meat types were not indicated on product labels (n=21).

Sample Number	Genus (% Of Average Fold)
**15**©	*Bos* (Cattle) (**37,1**) *Sus* (Pig) (38,5), *Ovis* (Sheep) (23,7) and *Rupicapra* (Goat Antelope) (0,2)
**159**©	*Ovis* (Sheep) (**91,4**), *Bos* (Cattle) (7,0), *Bison* (Bison) (0,1), *Rupicapra* (Goat Antelope) (1,3) and *Naemorhedus* (Goral) (0,1)
16	*Bos* (Cattle) (99,5), *Bubalus* (Buffalo) (0,2), *Rupicapra* (Goat Antelope) (0,1) and *Redunca* (Reedbuck) (0,1)
18	*Bos* (Cattle) (99,3), *Bubalus* (Buffalo) (0,2), *Gallus* (Chicken) (0,2), *Sus* (Pig) (0,1), *Rupicapra* (Goat Antelope) (0,1) and *Redunca* (Reedbuck) (0,1)
**26**©	Bos (Cattle) (**91,0**), *Ovis* (Sheep) (5,2), *Sus* (Pig) (3,0), *Bubalus* (Buffalo) (0,3), *Rupicapra* (Goat Antelope) (0,2) and *Redunca* (Reedbuck) (0,1)
27	*Bos* (Cattle) (99,5), *Sus* (Pig) (0,1), *Bubalus* (Buffalo) (0,1) and *Rupicapra* (Goat Antelope) (0,1)
29	*Bos* (Cattle) (98,8), *Ovis* (Sheep) (0,6), *Bubalus* (Buffalo) (0,2), *Sus* (Pig) (0,1), *Rupicapra* (Goat Antelope) (0,1) and *Redunca* (Reedbuck) (0,1)
38	Bos (Cattle) (99,0), *Bubalus* (Buffalo) (0,3), *Rupicapra* (Goat Antelope) (0,2), *Ovis* (Sheep) (0,1) and *Redunca* (Reedbuck) (0,1)
**4**©	*Sus* (Pig) (**97,4**) and *Bos* (Cattle) (2,7)
42	*Bos* (Cattle) (99,3), *Sus* (Pig) (0,3), *Bubalus* (Buffalo) (0,2), *Rupicapra* (Goat Antelope) (0,1) and *Redunca* (Reedbuck) (0,1)
43	*Bos* (Cattle) (99,5), *Bubalus* (Buffalo) (0,2), *Rupicapra* (Goat Antelope) (0,1), *Redunca* (Reedbuck) (0,1) and *Hemitragus* (Tahr) (0,1)

**5©**	*Sus* (Pig) (**94,8**) and *Bos* (Cattle) (5,2)
**68©**	*Bos* (Cattle) (**97,9**), *Bubalus* (Buffalo) (0,8), *Rupicapra* (Goat Antelope) (0,6), *Redunca* (Reedbuck) (0,3) and *Bison* (Bison) (0,2)
7	*Bos* (Cattle) (99,6), *Bubalus* (Buffalo) (0,2), *Ovis* (Sheep) (0,1), *Bison* (Bison) (0,1), *Rupicapra* (Goat Antelope) (0,1) and *Redunca* (Reedbuck) (0,1)
72	*Bos* (Cattle) (98,9), *Bubalus* (Buffalo) (0,5), *Rupicapra* (Goat Antelope) (0,3) and *Redunca* (Reedbuck) (0,2)
75	*Bos* (Cattle) (99,2), *Bubalus* (Buffalo) (0,3), *Sus* (Pig) (0,2), *Rupicapra* (Goat Antelope) (0,2) and *Redunca* (Reedbuck) (0,1)
**79©**	*Bos* (Cattle) (**95,9**), *Ovis* (Sheep) (3,6), *Rupicapra* (Goat Antelope) (0,2), *Bubalus* (Buffalo) (0,1), *Bison* (Bison) (0,1) and *Redunca* (Reedbuck) (0,1)
82	*Bos* (Cattle) (99,5), *Sus* (Pig) (0,1), *Ovis* (Sheep) (0,1), *Bubalus* (Buffalo) (0,1) and *Rupicapra* (Goat Antelope) (0,1)
**83©**	*Bos* (Cattle) (**74,7**), *Sus* (Pig) (24,7), *Ovis* (Sheep) (0,2), *Bubalus* (Buffalo) (0,2), *Rupicapra* (Goat Antelope) (0,1) and *Redunca* (Reedbuck) (0,1)
85	*Bos* (Cattle) (97,5), *Equus* (Horse/Donkey) (0,9), *Sus* (Pig) (0,8), *Ovis* (Sheep) (0,2), *Bubalus (*Buffalo) (0,2), *Rupicapra* (Goat Antelope) (0,2) and *Redunca* (Reedbuck) (0,1)
87	*Bos* (Cattle) (99,3), *Rupicapra* (Goat Antelope) (0,2), *Sus* (Pig) (0,1), *Bubalus* (Buffalo) (0,1) and *Redunca* (Reedbuck) (0,1)

Table S11. Percentage average folds of DNA from retail meat products labelled as beef sausage (N=21).

Sample Number	Genus (% Of Average Fold)
S	*Bos* (Cattle) (98,7), *Ovis* (Sheep) (0,5), *Sus* (Pig) (0,4) and *Homo* (Human) (0,1)
103	*Bos* (Cattle) (99,2), *Sus* (Pig) (0,2), *Bubalus* (Buffalo) (0,2), *Rupicapra* (Goat Antelope) (0,2), *Bison* (Bison) (0,1) and *Redunca* (Reedbuck) (0,1)
**104©**	*Bos* (Cattle) (**63,4**), *Sus* (Pig) (36,1), *Ovis* (Sheep) (0,1), *Bubalus* (Buffalo) (0,1) and *Rupicapra* (Goat Antelope) (0,1)
114	*Bos* (Cattle) (98,4), *Bubalus* (Buffalo) (0,6), *Rupicapra* (Goat Antelope) (0,6), *Redunca* (Reedbuck) (0,2) and *Bison* (Bison) (0,1)
**126©**	*Bos* (Cattle) (**78,1**), *Ovis* (Sheep) (20,8) and *Capra* (Goat) (1,0)
**127©**	*Bos* (Cattle) (**69,2**), *Sus* (Pig) (30,4), *Bubalus* (Buffalo) (0,1) and *Rupicapra* (Goat Antelope) (0,1)
129	*Bos* (Cattle) (98,8), *Ovis* (Sheep) (0,9), *Sus* (Pig) (0,1), *Bubalus* (Buffalo) (0,1) and *Rupicapra* (Goat Antelope) (0,1)
**134©**	*Bos* (Cattle) (**67,5**), *Ovis* (Sheep) (17,7), *Sus* (Pig) (14,3), *Rupicapra* (Goat Antelope) (0,3) and *Bubalus* (Buffalo) (0,1)
**135©**	*Bos* (Cattle) (**38,8**), *Sus* (Pig) (60,1), *Ovis* (Sheep) (0,8), *Homo* (Human) (0,2) and *Mus* (Rat) (0,2)
**143©**	*Bos* (Cattle) (**93,6**), *Ovis* (Sheep) (5,6), *Bubalus* (Buffalo) (0,2), *Rupicapra* (Goat Antelope) (0,2) and *Redunca* (Reedbuck) (0,1)
**144©**	*Bos* (Cattle) (**85,7**), *Ovis* (Sheep) (13,6), *Bubalus* (Buffalo) (0,2), *Rupicapra* (Goat Antelope) (0,2) and *Redunca* (Reedbuck) (0,1)
**145©**	*Bos* (Cattle) (**94,4**), *Sus* (Pig) (3,7), *Ovis* (Sheep) (0,9), *Bubalus* (Buffalo) (0,3), *Rupicapra* (Goat Antelope) (0,3) and *Redunca* (Reedbuck) (0,1)
**15©**	*Bos* (Cattle) (**18,1**), *Sus* (Pig) (78,1), *Ovis* (Sheep) (3,5) and *Rupicapra* (Goat Antelope) (0,2)
**155©**	*Bos* (Cattle) (**85,7**), *Sus* (Pig) (9,9), *Ovis* (Sheep) (4,0), *Bubalus* (Buffalo) (0,1) and *Rupicapra* (Goat Antelope) (0,1)
**156©**	*Bos* (Cattle) (**79,0**), *Sus* (Pig) (20,5), *Ovis* (Sheep) (0,1), *Bubalus* (Buffalo) (0,1), *Rupicapra* (Goat Antelope) (0,1) and *Redunca* (Reedbuck) (0,1)
174	*Bos* (Cattle) (99,5), *Ovis* (Sheep) (0,1), *Bubalus* (Buffalo) (0,1), *Rupicapra* (Goat Antelope) (0,1) and *Redunca* (Reedbuck) (0,1)
178	*Bos* (Cattle) (99,7) and *Bubalus* (Buffalo) (0,1)
**184©**	*Bos* (Cattle) (**68,7**), *Ovis* (Sheep) (27,9) and *Sus* (Pig) (3,2)

186	*Bos* (Cattle) (88,2), *Ovis* (Sheep) (11,0), *Bubalus* (Buffalo) (0,3), *Rupicapra* (Goat Antelope) (0,3) and *Redunca* (Reedbuck) (0,1)
**188**©	*Bos* (Cattle) (**64,2**), *Sus* (Pig) (35,3), *Bubalus* (Buffalo) (0,2) and *Rupicapra* (Goat Antelope) (0,1)
**189**©	*Bos* (Cattle) (**68,8**), *Sus* (Pig) (30,1), *Ovis* (Sheep) (0,8), *Bubalus* (Buffalo) (0,1) and *Rupicapra* (Goat Antelope) (0,1)
